# BMP-induced Atoh8 attenuates osteoclastogenesis by suppressing Runx2 transcriptional activity and reducing the Rankl/Opg expression ratio in osteoblasts

**DOI:** 10.1038/s41413-020-00106-0

**Published:** 2020-09-02

**Authors:** Yuhei Yahiro, Shingo Maeda, Masato Morikawa, Daizo Koinuma, Go Jokoji, Toshiro Ijuin, Setsuro Komiya, Ryoichiro Kageyama, Kohei Miyazono, Noboru Taniguchi

**Affiliations:** 1 Department of Orthopaedic Surgery, Kagoshima University, Kagoshima, Kagoshima 890-8520 Japan; 2 Department of Medical Joint Materials, Kagoshima University, Kagoshima, Kagoshima 890-8520 Japan; 3 Department of Bone and Joint Medicine, Kagoshima University, Kagoshima, Kagoshima 890-8520 Japan; 4 Department of Molecular Pathology, Graduate School of Medicine, The University of Tokyo, Bunkyo-ku, Tokyo, 113-0033 Japan; 5 Institute for Frontier Life and Medical Sciences, Kyoto University, Sakyo-ku, Kyoto, 606-8507 Japan

**Keywords:** Bone, Homeostasis

## Abstract

Adult bone structural integrity is maintained by remodeling via the coupling of osteoclastic bone resorption and osteoblastic bone formation. Osteocytes or osteoblasts express receptor activator of nuclear factor κ-B ligand (Rankl) or osteoprotegerin (Opg) to promote or inhibit osteoclastogenesis, respectively. Bone morphogenetic protein (BMP) is a potent bone inducer, but its major role in adult bone is to induce osteocytes to upregulate sclerostin (Sost) and increase the Rankl/Opg expression ratio, resulting in promotion of osteoclastogenesis. However, the precise effect of BMP-target gene(s) in osteoblasts on the Rankl/Opg expression ratio remains unclear. In the present study, we identified atonal homolog 8 (*Atoh8*), which is directly upregulated by the BMP-Smad1 axis in osteoblasts. In vivo, Atoh8 was detected in osteoblasts but not osteocytes in adult mice. Although global Atoh8-knockout mice showed only a mild phenotype in the neonate skeleton, the bone volume was decreased and osteoclasts were increased in the adult phase. Atoh8-null marrow stroma cells were more potent than wild-type cells in inducing osteoclastogenesis in marrow cells. Atoh8 loss in osteoblasts increased Runx2 expression and the Rankl/Opg expression ratio, while Runx2 knockdown normalized the Rankl/Opg expression ratio. Moreover, Atoh8 formed a protein complex with Runx2 to inhibit Runx2 transcriptional activity and decrease the Rankl/Opg expression ratio. These results suggest that bone remodeling is regulated elaborately by BMP signaling; while BMP primarily promotes bone resorption, it simultaneously induces Atoh8 to inhibit Runx2 and reduce the Rankl/Opg expression ratio in osteoblasts, suppressing osteoclastogenesis and preventing excessive BMP-mediated bone resorption.

## Introduction

In adult bone, bone remodeling maintains structural integrity via the clearance and repair of damaged bone and regulates mineral homeostasis.^1,2^ Osteoclastic bone resorption and osteoblastic bone formation occur sequentially in a synchronized manner at adjacent anatomical spots to preserve bone volume (BV).^3,4^ The differentiation of osteoblasts is governed by the master regulator transcription factor Runx2.^5^ Runx2 upregulates osteoblast-specific genes, such as osterix (Osx; *Sp7*), alkaline phosphatase (ALP; *Alpl*), type I collagen (*Col1a1*), bone sialoprotein (Bsp; *Ibsp*), and osteocalcin (Ocn; *Bglap*).^6^ Mononuclear cells originating from hematopoietic stem cells differentiate into multinucleated osteoclasts. Macrophage colony-stimulating factor (M-CSF) produced by osteoblasts^7^ and receptor activator of nuclear factor κ-B ligand (Rankl; *Rankl*) secreted by bone marrow stromal cells, osteoblasts, and osteocytes are crucial for inducing osteoclast differentiation.^8^ Conversely, osteoblasts and stromal cells secrete osteoprotegerin (Opg; *Opg*), which binds to Rankl as a decoy receptor to block the interaction of Rankl with receptor activator of nuclear factor κ-B (Rank), to inhibit osteoclastogenesis.^9^ Therefore, the Rankl/Opg expression ratio in bone is considered a marker of osteoclast activation status.

Bone morphogenetic proteins (BMPs), potent bone-inducing factors^10^, are members of the transforming growth factor-β (TGF-β) family, which includes multifunctional regulators involved in various cellular processes, such as proliferation, migration, apoptosis, and differentiation.^11^ BMPs bind to type I and II receptors on the cell membrane to phosphorylate and activate receptor-regulated Smads (R-Smads), Smad1 and Smad5, in the cytoplasm. Two BMP-activated R-Smad molecules form a trimer with Smad4, a common partner Smad. Next, these Smad complexes translocate into the nucleus and activate the expression of target genes as transcription factor complexes.^11^ BMP signaling promotes osteoblast differentiation; BMP-activated Smads induce expression of Runx2, which, in turn, forms a complex with Runx2 to initiate osteoblast-specific gene expression.^12–14^ During bone remodeling, BMP signaling positively regulates osteoclastogenesis by supporting Rankl expression in bone marrow stromal cells, osteoblasts, and osteocytes. Therefore, the ablation of BMP signaling in these cells in adult mice results in increased bone mass due to a significant decrease in bone resorption, with a decreased Rankl/Opg expression ratio in bone.^15–19^ Mechanistically, BMP signaling stimulates osteocytes to express sclerostin (Sost), a canonical Wnt pathway inhibitor.^19,20^ Because Wnt signaling inhibits the Rankl/Opg expression ratio in osteoblasts,^20^ the major role of BMP signaling in adult bone remodeling is believed to be to increase the Rankl/Opg expression ratio in mature osteoblasts or osteocytes, at least in part by inducing Sost expression, thereby promoting osteoclastic bone resorption. Provided that Sost and Rankl are primarily and strongly expressed in mechanosensing osteocytes, which are embedded in bone,^21,22^ the precise effects of BMP signaling and its target gene(s) on immature osteoblasts, such as marrow stromal cells, on regulation of the Opg/Rankl expression ratio are still unclear.

In this study, we performed a microarray screen by stimulating the mouse bone marrow stromal cell line ST-2 with BMP-2 and identified atonal homolog 8 (*Atoh8*) as a direct target gene of the BMP-Smad pathway. To determine the effects of *Atoh8*, we studied the bones of global Atoh8-knockout (KO) adult mice by microcomputed tomography (μ-CT) and histomorphometric analysis and evaluated the phenotypes of primary cells. We found that Atoh8 inhibits Runx2 function and reduces the Rankl/Opg expression ratio in osteoblasts to prevent bone loss.

## Results

### The BMP-Smad1 pathway directly induces Atoh8 expression in differentiating osteoblasts

**Fig. 1:**
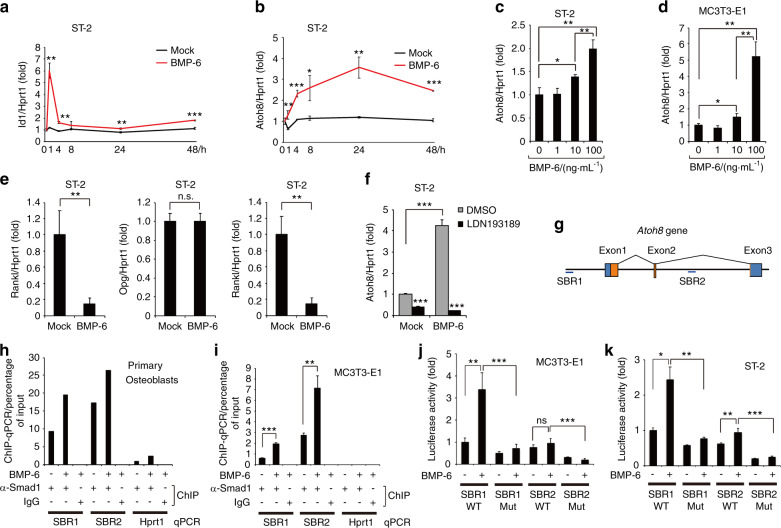
The BMP-6-Smad1 pathway directly induces Atoh8 expression in osteoblasts. **a**–**e** ST-2 stromal cells or MC3T3-E1 osteoblasts were stimulated with 100 ng·mL^–1^ or the indicated concentrations of BMP-6 for 48 h and subjected to RT-qPCR for *Id1*, *Atoh8*, *Rankl*, and *Opg*. Values were normalized to *Hprt1* (*n* = 3). **f** ST-2 cells were stimulated with 100 ng·mL^–1^ BMP-6 with or without 1 μmol·L^–1^ LDN193189 for 48 h and subjected to RT-qPCR for *Atoh8*. Values were normalized to *Hprt1* (*n* = 3). **g** Structure of murine *Atoh8*, including Smad1/5-binding regions (SBRs), is illustrated. Blue boxes, untranslated regions; orange boxes, protein-coding regions. ChIP was performed using an anti-Smad1 antibody and lysate of mouse primary osteoblasts (*n* = 1) (**h**) or MC3T3-E1 osteoblasts (*n* = 3) (**i**), and purified DNA fragments were subjected to qPCR with primer sets against the indicated regions. The Hprt1 gene served as a negative control. MC3T3-E1 (**j**) or ST-2 (**k**) cells were transfected with the indicated reporter constructs, stimulated with 100 ng·mL^–1^ BMP-6, and subjected to a luciferase assay (*n* = 3). Data are shown as the mean ± SD. n.s., not significant; *P < 0.05; **P < 0.01; ***P < 0.001

ST-2 cells are osteoblastic stromal cells that have been widely used for the analysis of bone remodeling in vitro.^23,24^ ST-2 cells can induce osteoclastogenesis of murine bone marrow cells in a coculture system,^25^ while BMP-2, BMP-4, and BMP-6 promote the osteoblastic differentiation of ST-2.^26,27^ The loss of BMP signaling in ST-2 cells in mice resulted in reduced Rankl expression.^15^ To search for BMP-target genes in differentiating stromal cells that may affect the Rankl/Opg expression ratio, we stimulated ST-2 cells with BMP-2 for 48 h to induce osteoblastic differentiation and extracted messenger RNA (mRNA) to perform microarray analysis. After excluding genes with signal intensities < 50, which were considered not to be functional because of low expression levels, we purified the top 10 genes upregulated by BMP-2 (Table 1). The expression of representative osteoblast-specific genes, *Ibsp*, *Sp7*, and *Alpl*, increased, confirming that osteoblast differentiation proceeds under these conditions. We focused on *Atoh8* because it is the only gene that has not been reported to be associated with osteoblast biology. During the process of bone remodeling, osteoclasts secrete BMP-6 to promote osteoblastic bone formation.^28^ Moreover, because BMP-6 is more efficient in bone formation than BMP-2 in MSCs,^29^ we used BMP-6 to investigate the expression and function of Atoh8 in the following experiments. By reverse transcription-quantitative polymerase chain reaction (RT-qPCR), we confirmed that the application of 100 ng·mL^−1^ exogenous BMP-6 induced a transient increase in Id1 expression at 1 h in ST-2 cells, showing that the canonical BMP-Smad1/5 pathway was stimulated (Fig. 1a).^30^ In contrast, Atoh8 gradually accumulated from 4 h and was continuously elevated until 48 h after BMP-6 induction (Fig. 1b). This effect of BMP-6 on Atoh8 expression was dependent on dose. Although BMP-6 exerted marginal effects at 10 ng·mL^−1^, it significantly increased Atoh8 expression at 100 ng·mL^−1^ in both ST-2 stromal cells and MC3T3-E1 osteoblasts at 48 h (Fig. 1c, d). During this period, Rankl expression was dramatically diminished, while Opg expression remained unchanged, resulting in a markedly decreased Rankl/Opg expression ratio (Fig. 1e). We found that LDN193189, a specific inhibitor of BMP type I receptors, strongly blocked the BMP-6-induced increase in Atoh8 expression and that it also reduced the basal level of Atoh8 in the absence of exogenous BMP-6 (Fig. 1f), suggesting that Atoh8 induction was dependent on BMP signaling and that endogenous BMPs and/or BMPs in the supernatant of culture medium induced measurable levels of Atoh8. We previously performed chromatin immunoprecipitation sequencing (ChIP-seq) analysis using human umbilical vein endothelial cells stimulated with BMP-9 to map Smad1/5 occupancy on the genome and found that the *Atoh8* gene locus was bound by an anti-Smad1/5 antibody.^31^ To test this in osteoblasts, we conducted a ChIP-qPCR assay. We identified two putative Smad1/5-binding regions (SBRs) in the murine *Atoh8* gene locus, one 10 kb upstream of the transcription start site (GACGCC: SBR1) and the other in the second intron (GGCGCC: SBR2) (Fig. 1g); both regions are well conserved from zebrafish to humans.^32^ In primary osteoblasts purified from newborn mouse calvariae or MC3T3-E1 osteoblasts, the anti-Smad1 antibody immunoprecipitated fragments of SBR1 and SBR2; while SBR2 signals were predominant, both SBRs were further enriched upon BMP-6 treatment (Fig. 1h, i). To evaluate the potential of SBRs as transcriptional enhancers in osteoblasts, we used luciferase reporter constructs of SBR1 or SBR2^32^ and performed luciferase assays. BMP-6 enhanced SBR1 activity in both MC3T3-E1 and ST-2 cells, while it only faintly enhanced SBR2 (Fig. 1j, k). BMP-induced activation and steady-state activity were eliminated by mutations in SBRs (Fig. 1j, k), indicating that the transcriptional activity of SBRs depends on the specific Smad-binding sequence. These results demonstrated that Atoh8 expression is directly induced by BMP-activated Smad1 in differentiating osteoblastic cells.

**Table 1:** Top 10 upregulated genes 48 h after BMP-2 stimulation in ST-2 cells; microarray analysis

Mock_(signal)_	BMP-2_(signal)_	Fold upregulated by BMP-2	Genbank	Gene description	Gene symbol
85.876 47	579.827 33	6.751 876 62	AF172286	Hairy/enhancer-of-split related with YRPW motif 1	*Hey1*
191.642 85	1 151.693	6.009 579 799	BC045143	Integrin binding sialoprotein	*Ibsp*
73.915 56	309.060 33	4.181 262 105	BC057090	Fatty acid binding protein 7, brain	*Fabp7*
202.033 02	839.567 14	4.155 593 675	BC064779	Fibromodulin	*Fmod*
60.419 93	197.078 6	3.261 814 438	AF184902	Osterix	*Sp7*
331.555 82	1 063.337	3.207 113 059	BC013268	Wnt inhibitory factor 1	*Wif1*
100.814 48	291.313 17	2.889 596 514	BC065175	Alkaline phosphatase, liver/bone/kidney	*Alpl*
328.455 32	944.503 54	2.875 592 151	AB046527	Atonal homolog 8	*Atoh*8
381.240 17	980.749 15	2.572 523 116	BC115642	Kazal-type serine peptidase inhibitor domain 1	*Kazald1*
336.318 97	836.593 26	2.487 499 471	AB007848	Osteomodulin	*Omd*

### Atoh8 is expressed in osteoblasts in adult bone

**Fig. 2:**
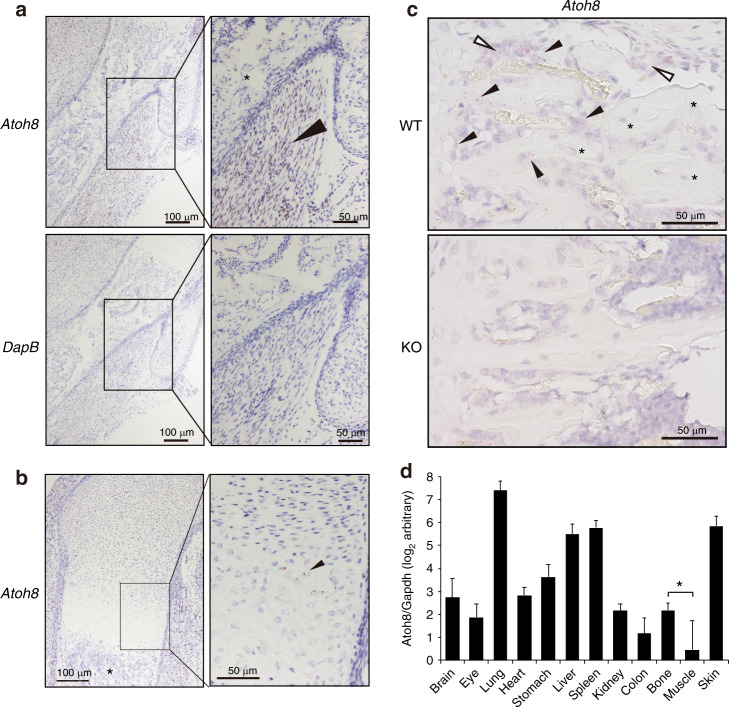
Atoh8 is absent in embryonic bone osteoblasts but expressed in prehypertrophic chondrocytes, and it is detected in osteoblasts of adult bone. **a** ISH analysis of the mouse E17.5 humerus with the *Atoh8* probe. A *DapB* probe served as a negative control. Brown positive signals of Atoh8 were evident in muscle (arrowhead) but absent in bone osteoblasts (asterisk). Scale bar = 100 μm (left panels) and 50 μm (right panels). **b** ISH analysis of the mouse E17.5 humerus with the *Atoh8* probe. Red positive signals of Atoh8 were detected in prehypertrophic chondrocytes (arrowhead) but absent in bone osteoblasts (asterisk). Scale bar = 100 μm (left panel) and 50 μm (right panel). **c** ISH analysis of the tibiae of 8-week-old mice with the *Atoh8* probe. A *Ppib* or a *DapB* probe served as the positive or a negative control, respectively (Fig. S1). Red positive signals of Atoh8 were evident in bone-lining osteoblasts (arrowheads) and clusters of stromal cells (open arrowheads) but absent in bone-embedded osteocytes (asterisks). WT wild type; KO knockout. Scale bar = 50 μm. **d** mRNA was purified from the indicated tissues collected from 8-week-old wild-type mice and subjected to RT-qPCR analysis of *Atoh8*. Values were normalized to *Gapdh* expression (*n* = 5). Data are shown as the mean of log_2_ value ± SD. *P < 0.05

To test whether Atoh8 is expressed in osteoblasts in vivo, we performed in situ hybridization (ISH) of *Atoh8* because of the lack of specific antibodies against murine Atoh8.^33^ In E17.5 mouse embryos, we successfully detected strong Atoh8 expression in skeletal muscles adjacent to bone (Fig. 2a, arrowhead), although no signals were detected by the negative control probe DapB. However, no signals were observed in osteoblasts (Fig. 2a, b, asterisks), and prehypertrophic chondrocytes exhibited substantial signal spots of Atoh8, as reported previously^34^ (Fig. 2b, arrowhead). In tibiae from 8-week-old adult mice, we detected appreciable expression of Atoh8 in clusters of stromal cells (Fig. 2c, open arrowheads) and osteoblasts lining the surface of trabecular bone of wild-type mice (Fig. 2c, arrowheads). Notably, bone-embedded osteocytes were negative for Atoh8 expression (Fig. 2c, asterisks). Recently, we generated a constitutive Atoh8-KO mouse (*Atoh8*^*LacZ(ex1)*^) by replacing a large part of exon 1 with a LacZ-reporter cassette.^32^ We confirmed that Atoh8 mRNA expression was absent in KO bone (Fig. 2c, lower panel). The validity of ISH was monitored by positive and negative control probes (Fig. S1). Next, to evaluate the tissue distribution of *Atoh8* gene expression, we examined the tissue RNA panel obtained from wild-type 8-week-old adult mice (Fig. 2d). Expression was predominantly observed in the lung, liver, spleen, and skin, while bone showed a substantial level of Atoh8 (Fig. 2d). Importantly, despite the strong expression of Atoh8 in embryonic muscle, it was weakly expressed in the muscle of adult mice (Fig. 2d).

### Atoh8 ablation in mice reduces adult bone volume

**Fig. 3:**
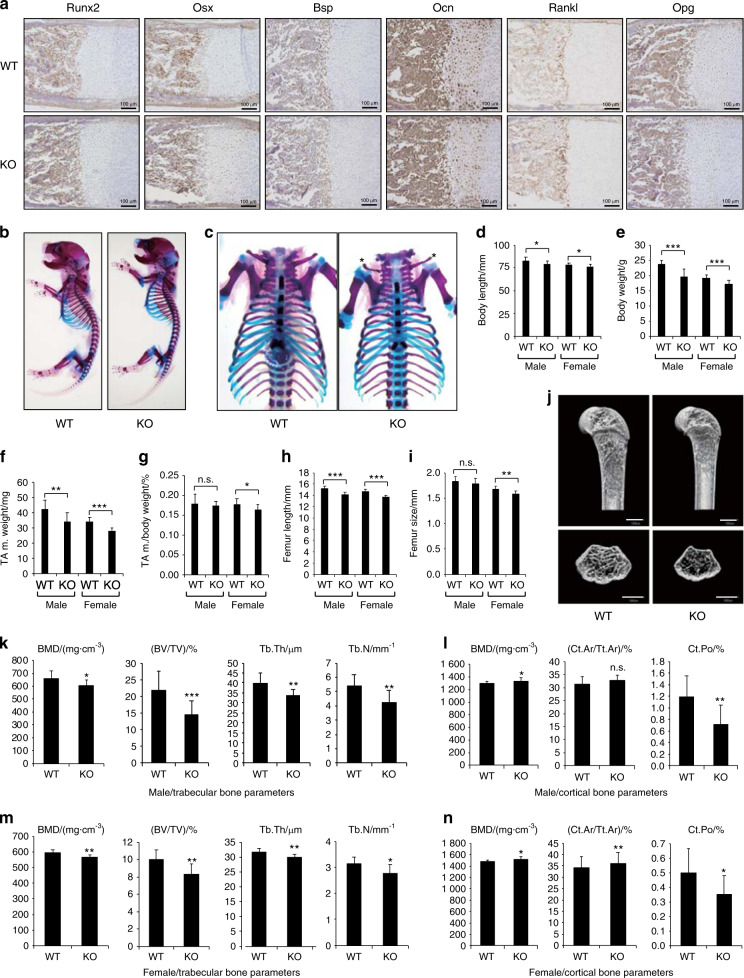
Atoh8-depleted adult mice exhibit reduced bone volume. **a** IHC using the indicated antibodies on E17.5 embryo humerus samples. Scale bar = 100 μm. **b, c** Skeletal preparation of newborn mice. Hypomorphic claviculae of mutant neonates are indicated (asterisks) (**c**). Body length (head to sacrum without tail) (**d**), body weight (**e**), tibialis anterior muscle weight (**f**), tibialis anterior muscle weight per body weight (**g**), femur length (**h**), and femur size (**i**) of 8-week-old mice were measured (*n* = 17 WT males, 11 KO males, 10 WT females, and 9 KO females). **j**–**n** μ-CT analysis of the femurs of 8-week-old male mice. Representative sagittal and axial sections of 3D μ-CT images are presented (**j**). Scale bar = 1 000 μm. Bone mineral density (BMD), bone volume per total volume (BV/TV), trabecular thickness (Tb.Th), and trabecular number (Tb.N) of the trabecular bone are shown (**k**, **m**). BMD, cortical bone area per total bone area (Ct.Ar/Tt.Ar), and cortical porosity (Ct.Po), of the cortical bone are shown (**l**, **n**). *n* = 15 WT males, 11 KO males, 10 WT females, and 9 KO females. Data are shown as the mean ± SD. n.s. not significant; *P < 0.05; **P < 0.01; ***P < 0.001

Our Atoh8-null mice were viable and obtained at the expected Mendelian ratio. Immunohistochemistry (IHC) analysis of humerus sections from E17.5 embryos demonstrated that osteoblastic markers, such as Runx2, Osx, Bsp, Ocn, and Rankl/Opg, were expressed normally in KO bone (Fig. 3a), suggesting that bone formation and osteoclast regulators were unaltered. As expected, skeletal preparations of our newborn mutant mice showed no obvious alterations in their size or proportion (Figs. 3b, c and S2). However, clavicles exhibited a thinned and shortened morphology (Figs. 3c and S2, asterisks). In our 8-week-old Atoh8-KO mice, body length was mildly reduced (Fig. 3d). Body weight was also decreased in mutant mice (Fig. 3e). We measured the weight of the tibialis anterior muscle and found that it was appreciably decreased in Atoh8-null mice (Fig. 3f). However, the ratio per body weight showed little change in Atoh8-KO mice (Fig. 3g), suggesting that this muscle decrease was a proportional systemic issue rather than a muscle-specific effect. Bone length and size were decreased measurably (Fig. 3h, i). Importantly, μ-CT analysis revealed that while bone size was reduced in KO mice as expected (Fig. 3j), bone mineral density (BMD), together with BV per total volume (BV/TV), trabecular thickness, and trabecular number, was also significantly reduced in cancellous bone of Atoh8-KO mice of both genders (Fig. 3k, m). Conversely, in cortical bone, BMD was increased mildly, with reduced bone porosity and a marginally enlarged cortical area (Fig. 3l, n). These phenotypes in adult KO mice were not specific to gender, negating the effect of sex hormones.

**Fig. 4:**
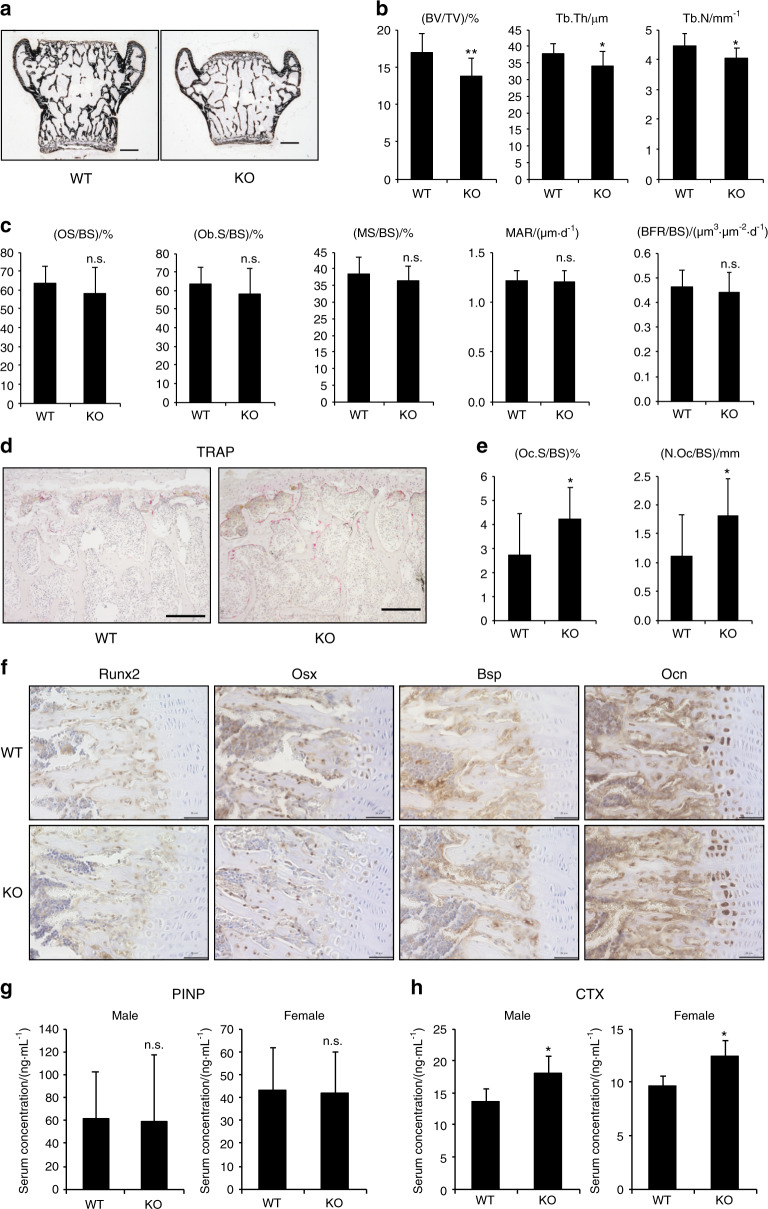
Osteoclastogenesis increases in Atoh8-KO bone to reduce bone volume. **a** Representative von Kossa staining images of coronal sections of lumbar vertebrae of 8-week-old male mice. Scale bar = 500 μm. **b**–**e** Bone histomorphometry analysis of lumbar vertebrae of 8-week-old male mice (*n* = 17 WT and 11 KO). Bone volume per total volume (BV/TV), trabecular thickness (Tb.Th), and trabecular number (Tb.N) are presented (**b**). Bone formation parameters are presented: osteoid surface/bone surface (OS/BS), osteoblast surface (Ob.S/BS), mineralizing surface (MS/BS), mineral apposition rate (MAR), and bone formation rate (BFR/BS) (**c**). Representative TRAP staining images of coronal sections of lumbar vertebrae (**d**). Scale bar = 200 μm. Osteoclast surface (Oc.S/BS) and osteoclast number (N.Oc/BS) are presented (**e**). **f** IHC of tibial samples from 8-week-old mice using the indicated antibodies. Scale bar = 50 μm. Enzyme-linked immunosorbent assays were performed to quantitate the N-terminal propeptide of type I procollagen (PINP) (**g**) and C-terminal telopeptides of type I collagen (CTX-I) (**h**) in the serum of 8-week-old mice (*n* = 4 WT males, 4 KO males, 3 WT females, and 4 KO females). Data are shown as the mean ± SD. n.s. not significant; *P < 0.05; **P < 0.01

To investigate whether bone formation or resorption was responsible for the reduced bone mass in Atoh8-KO mice, we conducted bone histomorphometric analysis. von Kossa staining of the coronal section of the lumbar vertebral body demonstrated decreased size and volume of bone (Fig. 4a), and BV/TV, trabecular thickness, and trabecular number were reduced in Atoh8-KO vertebrae (Fig. 4b). However, none of the bone formation parameters, i.e., osteoid surface (OS) per bone surface (BS), osteoblast surface (Ob.S), mineralizing surface (MS), mineral apposition rate (MAR), and bone formation rate (BFR) were affected by *Atoh8* loss (Fig. 4c). Conversely, osteoclasts, visualized by tartrate-resistant acid phosphatase (TRAP) staining, were increased (Fig. 4d), and the osteoclast surface (Oc.S) and number (N.Oc) were notably augmented in Atoh8-KO vertebrae (Fig. 4e). We also performed bone histomorphometric analysis of long bones (tibiae) and confirmed that essentially the same alterations were observed in adult KO mice of both genders (Fig. S3). To further check the status of bone formation in adult Atoh8-deficient mice, we evaluated the expression of osteoblastic marker proteins in tibiae by IHC, which revealed no significant alterations (Fig. 4f). Moreover, the serum concentration of N-terminal propeptide of type I procollagen (PINP), a bone formation marker, was not altered in adult KO mice (Fig. 4g). However, C-terminal telopeptides of type I collagen (CTX-I), a bone resorption marker, were augmented significantly in the serum of KO mouse regardless of gender (Fig. 4h). Taken together, these results demonstrate that bone resorption predominates in bone formation to reduce bone mass in Atoh8-KO mice.

### Atoh8 loss in stromal osteoblasts increases bone marrow cell osteoclastogenesis

**Fig. 5:**
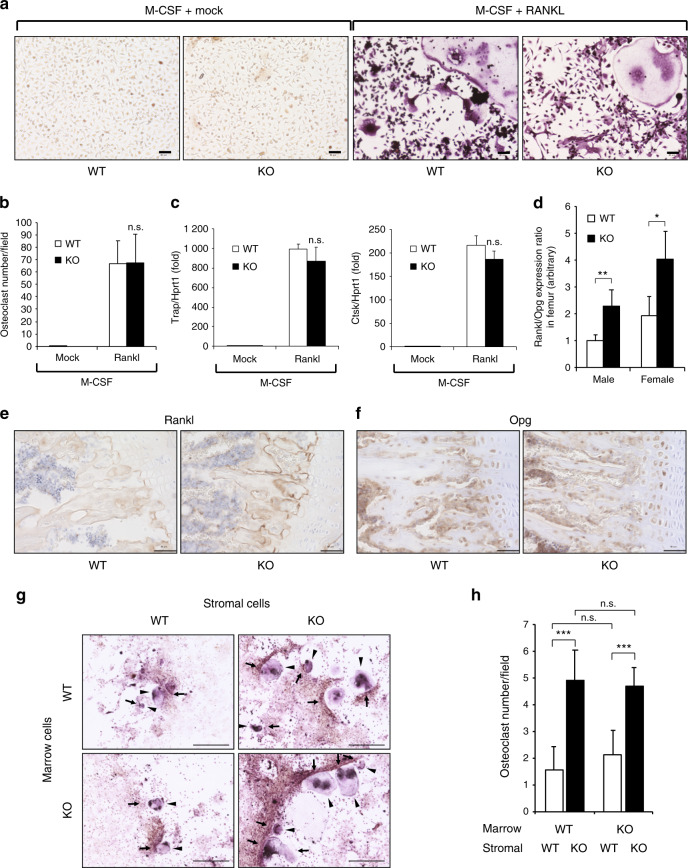
Atoh8-deficient bone exhibits a higher Rankl/Opg expression ratio, and marrow stromal cells are more potent than WT cells in inducing osteoclastogenesis of bone marrow cells. **a**–**c** Primary bone marrow cells of 8-week-old mice were stimulated with 50 ng·mL^−1^ M-CSF with or without 100 ng·mL^−1^ RANKL for osteoclastogenesis. Representative images of TRAP staining-positive osteoclasts are presented (**a**). Scale bar = 50 μm. The number of multinucleated (>3 nuclei) osteoclasts was counted in six fields for each sample (**b**). Cell lysates were subjected to RT-qPCR for *Trap* and *Ctsk* (**c**) (*n* = 3). **d** RNA was extracted from femurs of 8-week-old male mice and subjected to RT-qPCR to measure the Rankl/Opg expression ratio (*n* = 5 WT males, 4 KO males, 4 WT females, and 3 KO females). IHC of tibial samples from 8-week-old mice using anti-Rankl (**e**) and anti-Opg (**f**) antibodies. Scale bar = 50 μm. **g**, **h** Coculture of primary bone marrow stromal cells and bone marrow cells of the indicated genotype to induce osteoclastogenesis. TRAP staining-positive osteoclasts are indicated (arrowheads) (**g**). Scale bar = 500 μm. The number of multinucleated (>3 nuclei) osteoclasts was counted in nine fields for each sample (**h**). The data shown are the mean ± SD. n.s. not significant; *P < 0.05; **P < 0.01; ***P < 0.001

To evaluate whether enhanced osteoclastogenesis induced by Atoh8 defects in bone is an osteoclast-autonomous event and/or is indirectly mediated by osteoblastic coupling, we induced osteoclast differentiation of primary bone marrow cells by adding M-CSF and RANKL in vitro. The results showed that osteoclastogenesis was not affected by the *Atoh8* genotype (Fig. 5a); neither the number of generated osteoclasts (Fig. 5b) nor the expression of the osteoclast-specific genes *Trap* and *Ctsk* (Fig. 5c) were affected by Atoh8 deletion in bone marrow cells. In contrast, the Rankl/Opg expression ratio was significantly increased in femurs from adult Atoh8-KO mice of both genders, as evaluated by RT-qPCR (Fig. 5d). This alteration was confirmed by IHC analysis, showing that the expression of Rankl, but not Opg, was enhanced in the tibiae of adult KO mice (Fig. 5e, f). Next, to evaluate the role of stromal osteoblasts, we induced osteoclast differentiation of primary bone marrow cells by coculture with primary bone marrow stromal cells (Fig. 5g). Again, the genotype of bone marrow cells did not affect the number of differentiated osteoclasts (Fig. 5g, h). However, Atoh8*-*KO bone marrow stromal cells doubled osteoclastogenesis regardless of marrow cell genotype (Fig. 5g, h), suggesting that the increased Rankl/Opg expression ratio in Atoh8*-*KO bone marrow stromal cells promoted osteoclast differentiation.

Skeletal muscle mass is linked to bone mass at adult stages in which various muscle-derived humoral factors, such as myostatin, Fam5c, Irisin, Osteoglycin, Osteonectin, and Fgf2, affect osteoblastic bone formation (reviewed in)^35^. Both muscle mass and BV were reduced in our Atoh8-KO adult mice. Therefore, we detected the expression of these muscle-derived factors in the gastrocnemius muscle of KO mice by RT-qPCR. However, the expression levels of all tested genes were identical between the wild-type and KO samples (Fig. S4).

### Atoh8 loss promotes BMP-induced osteoblast differentiation, increases the Rankl/Opg expression ratio, and induces maturation into osteocytes in vitro

**Fig. 6:**
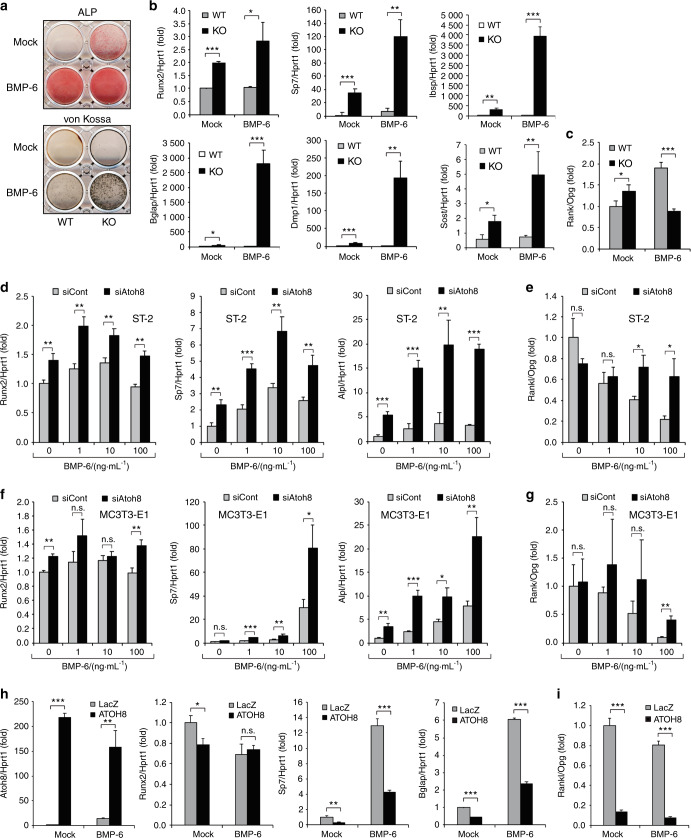
Atoh8 loss in osteoblasts accelerates Runx2 expression, osteoblastic differentiation, and the Rankl/Opg expression ratio. **a**–**c** Mouse primary osteoblasts were stimulated with or without 100 ng·mL^−1^ BMP-6 and subjected to ALP staining on day 4, von Kossa staining on day 11, and RT-qPCR on day 6 for the indicated genes (*n* = 3). Values were normalized to *Hprt1*. ST-2 (**d**, **e**) or MC3T3-E1 (**f**, **g**) cells were transfected with the indicated siRNA, stimulated with or without the indicated concentrations of BMP-6 for 2 days, and subjected to RT-qPCR for the indicated genes. Values were normalized to *Hprt1* (*n* = 3). **h**, **i** ST-2 cells were transfected with the indicated expression plasmid, stimulated with or without 100 ng·mL^−1^ BMP-6 for 2 days, and subjected to RT-qPCR. Values were normalized to *Hprt1* (*n* = 3). The data shown are the mean ± SD. n.s. not significant; *P < 0.05; **P < 0.01; ***P < 0.001

Next, we investigated the cell autonomous role of Atoh8 in osteoblastic differentiation. In Atoh8-KO primary osteoblast culture, ALP activity was enhanced, even in the absence of BMP-6, while bone nodule formation, evaluated by von Kossa staining, was strongly promoted by BMP-6 application (Fig. 6a). The expression of Runx2 and other osteoblast-specific genes (*Sp7*, *Ibsp*, and *Bglap*) was dramatically increased in Atoh8-KO osteoblasts in both BMP-6-treated and BMP-6-nontreated conditions (Fig. 6b), suggesting that Atoh8 inhibits Runx2 expression and osteoblast maturation, while endogenous BMPs or BMPs in the supernatant of culture medium induced substantial Atoh8 expression. Notably, the osteocyte-specific genes *Dmp1* and *Sost* were significantly augmented by BMP-6 in Atoh8-KO osteoblasts (Fig. 6b), indicating that these cells matured into osteocytes embedded in bone nodules. Atoh8 loss in immature osteoblasts without BMP-6 treatment (mock control) increased the Rankl/Opg expression ratio, while BMP-6-stimulated osteocytic cells showed a decreased Rankl/Opg expression ratio (Fig. 6c). To evaluate the effects of Atoh8 in more immature bone marrow stromal cells, we transfected Atoh8 short interfering RNA (siRNA) into ST-2 cells and induced differentiation with various concentrations of BMP-6. While 10 ng·mL^−1^ BMP-6 exhibited maximum induction of differentiation, loss of Atoh8 enhanced the expression of Runx2, Sp7, and Alpl regardless of BMP-6 concentration in ST-2 cells (Fig. 6d). Interestingly, however, BMP-6 suppressed the Rankl/Opg expression ratio in a dose-dependent manner, and this decline was prevented by siAtoh8 (Fig. 6e). In MC3T3-E1 osteoblasts, BMP-6 promoted differentiation and decreased the Rankl/Opg ratio in a dose-dependent manner, whereas the enhancing effect of siAtoh8 was maximized at 100 ng·mL^−1^ BMP-6 (Fig. 6f, g). Next, we transfected the human ATOH8 plasmid into ST-2 cells to achieve sufficient expression (Fig. 6h). ATOH8 mildly inhibited Runx2 expression in mock-treated ST-2 cells, while it significantly diminished the osteoblastic marker genes *Sp7* and *Bglap*, confirming that Atoh8 suppresses osteoblast differentiation in vitro (Fig. 6h). The ATOH8 expression vector drastically decreased the Rankl/Opg expression ratio, regardless of BMP-6 application (Fig. 6i). These results suggested that BMP-induced Atoh8 suppresses the differentiation of immature osteoblasts and reduces the Rankl/Opg expression ratio in vitro.

### Runx2 co-knockdown in ST-2 cells prevents Atoh8 siRNA-mediated enhancement of the Rankl/Opg expression ratio

**Fig. 7:**
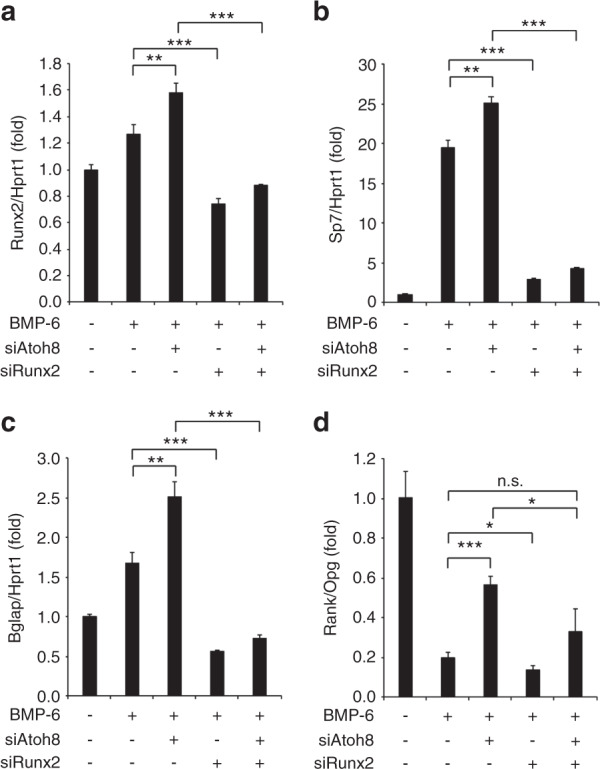
Atoh8 silencing-mediated promotion of Runx2 expression, osteoblast differentiation, and Rankl/Opg expression is inhibited by Runx2 knockdown in ST-2 cells. **a**–**d** ST-2 cells were transfected with the indicated siRNA, stimulated with or without 100 ng·mL^−1^ BMP-6 for 2 days, and subjected to RT-qPCR. Values were normalized to *Hprt1* (*n* = 3). The data shown are the mean ± SD. n.s. not significant; *P < 0.05; **P < 0.01; ***P < 0.001

Because Runx2 overexpression in osteoblasts reportedly promotes Rankl expression and inhibits Opg levels to enhance osteoclastogenesis,^36,37^ we tested whether increased Runx2 is responsible for the enhancement of the Rankl/Opg expression ratio by Atoh8 loss. In ST-2 cells, Runx2 knockdown inhibited the Atoh8 siRNA-mediated increase in Runx2 levels and subsequent Sp7 and Bglap upregulation, as expected (Fig. 7a–c). BMP-6 application decreased the Rankl/Opg expression ratio, which was recovered by siAtoh8 transfection. Moreover, this recovery by siAtoh8 was successfully prevented by Runx2 knockdown (Fig. 7d).

### Atoh8 forms a complex with Runx2 to interfere with Runx2 transcriptional activity

**Fig. 8:**
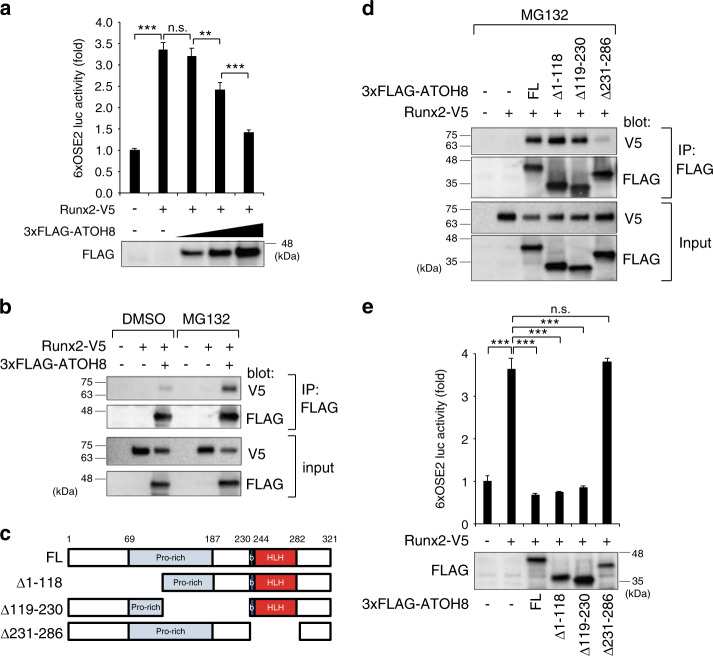
Atoh8 interacts with Runx2 and inhibits Runx2 transcriptional activity via the bHLH domain. **a** COS-7 cells were transfected with the indicated expression plasmids and subjected to the 6xOSE2 reporter luciferase assay (*n* = 3). The protein expression level of the FLAG-tagged Atoh8 vector was monitored by immunoblotting using an anti-FLAG antibody. **b** COS-7 cells were transfected with the indicated expression plasmids with or without 20 μmol·L^−1^ MG132 for 2 h and subjected to immunoprecipitation with the anti-FLAG antibody, followed by immunoblotting analysis with the indicated antibodies. **c** Schematic presentation of the constructs of full-length and deletion mutants of human ATOH8 used in this study. **d** COS-7 cells were transfected with the indicated expression plasmids with or without 20 μmol·L^−1^ MG132 for 2 h and subjected to immunoprecipitation with the anti-FLAG antibody, followed by immunoblotting analysis with the indicated antibodies. **e** COS-7 cells were transfected with the indicated expression plasmids and subjected to a 6xOSE2 reporter luciferase assay (*n* = 3). The protein expression level of the FLAG-tagged Atoh8 vectors was monitored by immunoblotting using the anti-FLAG antibody. The data shown are the mean ± SD. n.s. not significant; **P < 0.01; ***P < 0.001

Atoh8 depletion upregulated Runx2 and promoted differentiation in osteoblasts (Fig. 6b, d, f). Because Runx2 positively autoregulates its own promoter,^38^ enhanced Runx2 expression in Atoh8-deficient osteoblasts may be due to increased Runx2 transcriptional activity. To test this hypothesis, we used a reporter luciferase construct containing six tandem Runx2-binding elements, 6xOSE2.^39^ The Atoh8-expressing vector attenuated Runx2-induced luciferase activity in a dose-dependent manner (Fig. 8a). Immunoprecipitation assays with transfected expression plasmids revealed that Runx2 formed a complex with Atoh8 (Fig. 8b). This interaction was enhanced by treatment with the proteasome inhibitor MG132, suggesting that this protein complex is unstable and degraded by the proteasome. The *Atoh8* gene encodes a basic helix-loop-helix (bHLH) protein.^40^ To further confirm the specificity of this interaction, we used deletion mutant constructs of human ATOH8^32^ (Fig. 8c). Only the ATOH8 construct lacking the bHLH domain (231–286) did not form a complex with Runx2 (Fig. 8d). Last, we found that only the ATOH8 Δ231–286 mutant failed to suppress Runx2 transcriptional activity, as assessed by the 6xOSE2 reporter luciferase assay (Fig. 8e).

## Discussion

**Fig. 9:**
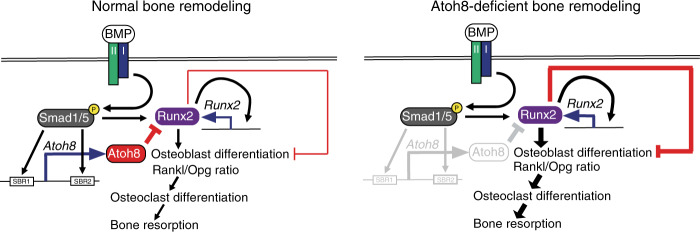
Illustration of this study’s results and a working model. BMP signaling enhances the Rankl/Opg expression ratio in cells of the osteoblastic lineage to promote osteoclastic bone resorption in bone remodeling. Moreover, the BMP-Smad pathway directly induces Atoh8 in immature osteoblasts. Atoh8 interacts with Runx2 and inhibits Runx2 transcriptional activity to suppress autoregulation of Runx2 and subsequent osteoblastic differentiation, as well as the Rankl/Opg expression ratio, thereby regulating the osteoclast number negatively to prevent excessive BMP-mediated bone resorption (left panel). The enhanced Runx2 activity in Atoh8-KO mice likely limited osteoblast maturation to maintain bone formation, while Runx2 increased the Rankl/Opg expression ratio to promote bone resorption and loss (right panel)

Figure 9 illustrates our results and working models. The BMP-Smad pathway directly induces Atoh8 in differentiating osteoblasts, and Atoh8 interacts with Runx2 to inhibit Runx2 transcriptional activity, thereby suppressing the autoregulation of Runx2 and subsequent osteoblastic differentiation (Fig. 9, left panel). In Atoh8-deficient mice, accelerated Runx2 activity promotes or suppresses osteoblast differentiation or maturation, respectively, maintaining bone formation (Fig. 9, right panel). Moreover, Atoh8 suppresses the Rankl/Opg expression ratio indirectly to regulate the osteoclast number and maintain the BV in adult mice, likely via inhibition of Runx2 activity, because Runx2 increases the Rankl/Opg expression ratio (Fig. 9, left panel).^37^ Thus, the loss of Atoh8 leads to decreased bone mass (Fig. 9, right panel).

Atoh8 is a mammalian homolog of the transcription factor atonal in which the bHLH domain is highly conserved across different species.^41^ In mouse embryos at E10.5–12.5, Atoh8 is expressed primarily in the retina, brain, lung, heart, liver, skeletal muscle, skin, and limb buds,^42^ while it is dynamically expressed in the retina and skeletal muscle, which is crucial for tissue development in zebrafish.^43^ Atoh8 also induces neurogenesis and inhibits gliogenesis during nervous system development.^40^ Some lines of *Atoh8*-deficient mice have been reported.^33,34,44–46^ These Atoh8-KO mice are viable and healthy and obtained at the expected Mendelian ratio.^33,34,45,46^ However, there is no report on the bone phenotype of adult Atoh8-deficient mice. In this study, we found ubiquitous expression of Atoh8 in tissues from adult mice, with high levels in the lung, stomach, liver, spleen, and skin (Fig. 2d). Therefore, the possible influence of non-bone tissue, such as cartilage and muscle, should be considered to interpret the bone phenotypes observed in our global Atoh8-KO mice. Recently, Schroeder et al. reported that Atoh8 is expressed in proliferating and hypertrophic chondrocytes, especially prehypertrophic chondrocytes, but not in osteoblasts of embryonic and developing mouse bone.^34^ Cartilage-specific Atoh8-KO mice showed delayed chondrocyte proliferation and differentiation in the short bones.^34^ Therefore, the decreased bone length in our Atoh8-KO adult mice might be the result of cartilage defects. However, the adult bone density appeared to be unaffected in cartilage-specific mutant mice.^34^ Atoh8 is expressed in a subset of embryonic muscle cells in the dermomyotome and myotome of chicken embryos^47^ and in human skeletal muscle during regeneration,^48^ and it plays roles in muscle differentiation and regeneration, whereas Atoh8 is downregulated during myogenic maturation of C2C12 cells in vitro.^47^ We also detected Atoh8 expression in embryonic muscle (Fig. 2a), but it was diminished in skeletal muscle of adult mice (Fig. 2d). Thus, Atoh8 expression was silenced in steady-state cells from mature adult muscle. Because the expression of muscle-derived bone regulators (e.g., myostatin, Fam5c, Irisin, Osteoglycin, Osteonectin, and Fgf2) may be changed in Atoh8-KO mice, we examined these factors by RT-qPCR but found no dysregulation in muscle from adult KO mice (Fig. S4). Overall, the possible effect of Atoh8 in muscle and cartilage on bone phenotypes, if there is any, can be considered minor.

The decrease in the Atoh8-null trabecular BV (Figs. 3 and 4) was contradictory to the in vitro phenotype of Atoh8-KO primary osteoblasts, which showed dramatically accelerated osteoblastic bone formation upon BMP-6 stimulation (Fig. 6). Most likely, the bone phenotype in Atoh8-KO mice can be explained by accelerated Runx2 activity induced by the loss of Atoh8. Runx2 is the osteoblast differentiation initiator that induces bone nodule formation in vitro. However, Runx2 inhibits osteoblast maturation in vivo, so bone-specific Runx2 transgenic adult mice show osteopenia, with increased bone resorption and normal or suppressed bone formation.^36,49^ Moreover, Runx2 is a promoter of the Rankl/Opg expression ratio.^37^ These data support our working model that loss of Atoh8 in mice promotes Runx2 activity, leading to an increased Rankl/Opg ratio and osteoclast differentiation, with no increase in trabecular bone formation. However, interestingly, in cortical bone, the mineral density was enhanced by Atoh8 deficiency, with significantly reduced porosity (Fig. 3l, n). Because mRNA extracted from total bone, a mixture of trabecular and cortical bones, showed an enhanced Rankl/Opg ratio in KO mice (Fig. 5d), it is likely that this increase in cortical bone mass was not the result of reduced bone resorption but increased bone formation, reflecting the osteoblast phenotype in vitro. Thus, the mode or extent of the Atoh8–Runx2 interaction may be different in cortical bone. It is unclear why the Rankl/Opg expression ratio was decreased in BMP-6-induced KO osteocytic cells, in contrast to our expectation (Fig. 6c); however, this phenotype is similar to that of Runx2 Tg primary osteoblasts; the Rankl/Opg expression ratio was enhanced in immature osteoblasts on day 3 of culture and suppressed in mature osteoblasts on day 10 of culture.^36^ Because it is difficult to fully examine the action of Atoh8 in bone remodeling using cultured osteoblasts in vitro and because Atoh8 is expressed ubiquitously in vivo, bone-specific inducible Atoh8-KO mice should be examined to reveal the precise role of Atoh8 in osteoblasts in the future.

Although we showed that Atoh8 is a direct target of the BMP-Smad pathway, the phenotype of Atoh8-null mice was inconsistent with those of multiple lines of BMP signaling component-depleted mice,^15–19^ indicating that Atoh8 is not a major target of the BMP pathway in bone remodeling but rather a common target of BMP and other signaling pathways. Indeed, the duration of Atoh8 increase upon BMP induction was significantly longer than that of the representative BMP-target gene Id1 (Fig. 1a, b), suggesting that other signaling pathway(s) maintained the expression at late timepoints. In our microarray analysis, *Hey1*, another bHLH transcription factor, which is a common direct target of BMP and Notch signaling, was the gene most elevated by BMP-2 stimulation in ST-2 cells (Table 1).^50,51^ In addition, Atoh8 is a target of Neurog3^44^ and NeuroD1.^52^ Therefore, other signaling systems, such as Notch or the Neurogenin/NeuroD family, are crucial in Atoh8 expression, in addition to BMP. In addition, a difference in Smad1 occupation behavior on SBR1 or SBR2 was observed between the ChIP-qPCR and luciferase assays. The SBR2 signal was predominant in ChIP, whereas SBR1 was predominant in the luciferase assay (Fig. 1h–k). ChIP assays can be influenced by factors bound on distant genomic regions in addition to the tested transcription factor, whereas luciferase activity reflects the interaction of only the tested protein on the limited short DNA fragment. Therefore, additional factor(s) bound outside of SBRs might be required to support Smad1 to activate SBR2. Thus, the possible complex regulation of Atoh8 expression may explain why it was not detected in either embryo bone osteoblasts or osteocytes in adult bone, in which BMP signaling is active (Fig. 2).

Atoh8 inhibits Runx2 transcriptional activity by forming a complex (Fig. 8). Some other bHLH transcription factors, such as Twist1/2^53^ and Hes1,^54^ bind to Runx2 to interfere with or promote its transcriptional activity, respectively. The mode of transcriptional regulation depends on other interacting proteins of corepressors or coactivators that constitute the complex. Atoh8 lacks a transactivation domain and possesses intrinsic repressor activity that depends on a conserved proline-rich domain, while it binds to the ubiquitous E protein E47 to repress E47 activity by preventing homodimerization and heterodimerization of E47 with other bHLH proteins.^55^ For Atoh8-mediated Runx2 inhibition, the proline-rich domain seems dispensable, while the bHLH domain is crucial (Fig. 8). Therefore, Atoh8 may cooperate with other bHLH factors, such as Twist proteins, to repress Runx2 transcriptional activity. We recently found that the half-life of exogenously expressed ATOH8 protein is ~30 min, while the ATOH8 mutant lacking the bHLH region (ATOH8 Δ231–286) is more stable than the full-length protein.^32^ Indeed, the Atoh8–Runx2 interaction is more evident in the presence of MG132 (Fig. 8b). These results suggested that Atoh8 is an unstable protein that is rapidly degraded in the proteasome and that the protein–protein interaction mediated by the bHLH domain affects the stability of Atoh8 and its binding partners, such as Runx2.

In conclusion, *Atoh8* is a novel BMP-target gene in osteoblasts that inhibits Runx2 transcriptional activity, the Rankl/Opg expression ratio and subsequent osteoclastogenesis, thereby preventing bone loss in mice. Thus, BMP signaling-mediated promotion of the Rankl/Opg expression ratio and bone resorption is counteracted by BMP-induced Atoh8 to tightly regulate bone remodeling in adult bone.

## Materials and methods

### Cell culture, reagents, and differentiation induction

The mouse bone marrow stromal cell lines ST-2 and COS-7 (used for in vitro transfection experiments) were obtained from the RIKEN BioResource Research Center (Tsukuba, Ibaraki, Japan). The mouse calvarial bone-derived osteoblast line MC3T3-E1 (clone 4) was obtained from the American Type Culture Collection (ATCC; Manassas, VA, USA). Primary C57BL/6J mouse osteoblasts were isolated and cultured as described previously.^56^ MC3T3-E1 cells and primary osteoblasts were cultured in minimum essential medium (MEM)-α (Gibco, Waltham, MA, USA). ST-2 cells were cultured in Roswell Park Memorial Institute 1640 medium (Gibco), and COS-7 cells were maintained in Dulbecco’s modified Eagle’s medium (Sigma-Aldrich, St. Louis, MO, USA).

For osteoblast differentiation, ST-2 cells were stimulated with recombinant human (rh) BMP-2 (Peprotech, Rocky Hill, NJ, USA) or rhBMP-6 (R&D Systems, Minneapolis, MN, USA) at the indicated concentrations. The BMP type I receptor inhibitor compound LDN193189 (Sigma-Aldrich) was dissolved in dimethyl sulfoxide (DMSO) to be added at 0.1 μmol·L^−1^, while 0.1% DMSO was used as the vehicle control. Osteoblast differentiation of primary osteoblasts was induced by 100 ng·mL^−1^ rhBMP-6, 0.05 mmol·L^−1^ ascorbic acid-2-phosphate (Sigma-Aldrich), and 10 mmol·L^−1^ β-glycerophosphate (Sigma-Aldrich). Primary bone marrow stromal cells and bone marrow osteoclast precursors were isolated from the femurs and tibiae of 8-week-old male C57BL/6J mice. The bones were dissected, and the bone marrow was flushed using phosphate-buffered saline (PBS). Cells were suspended in MEM-α medium supplemented with 1% sodium pyruvate and 0.02 mol·L^−1^ sodium bicarbonate. Adherent cells were used as stromal cells, nonadherent marrow cells in suspension were collected, and the cell pellet was incubated in red blood cell lysis buffer (Sigma-Aldrich; R7757). The remaining bone marrow cells were resuspended in culture medium supplemented with 50 ng·mL^−1^ rhM-CSF (R&D Systems) and incubated overnight to obtain adherent osteoclast precursors.

To induce osteoclastogenesis in bone marrow cells, adherent osteoclast precursors were plated at a density of 2.5–15 × 10^4^ cells per cm^2^ and supplemented with 50 ng·mL^−1^ rhM-CSF and 100 ng·mL^−1^ rhRANKL. Osteoclasts were observed on day 5. To generate osteoclasts by coculture of primary bone marrow stromal cells and bone marrow osteoclast precursors, bone marrow stromal cells were seeded at a density of 5 × 10^4^ cells in a 12-well plate in MEM-α medium supplemented with 10 nmol·L^−1^ 1,25 dihydroxyvitamin D_3_ (VD_3_; R&D Systems) and 1 μmol·L^−1^ prostaglandin E_2_ (PGE_2_; R&D Systems). The following day, 1 × 10^6^ bone marrow cells were seeded on stromal cells in MEM-α with VD_3_ and PGE_2_. Osteoclasts were present between days 7 and 9. All cell culture media contained 10% fetal bovine serum, 100 U·mL^−1^ penicillin G, and 100 μg·mL^−1^ streptomycin.

### Microarray analysis

ST-2 cells were incubated with 100 ng·mL^−1^ rhBMP-2 for 2 days before being lysed with TRIzol reagent (Invitrogen, Carlsbad, CA, USA) for mRNA purification. Then, mRNA samples were cleaned using the RNeasy MinqElute Cleanup kit (Qiagen, Hilden, Germany) and analyzed by Bio Matrix Research (Nagareyama, Chiba, Japan) using a Mouse Gene 2.0 ST Array (Affymetrix, Santa Clara, CA, USA).

### RT-qPCR

Cultured cells or minced bones were lysed using TRIzol reagent to purify RNA, and 1 µg of total RNA was subjected to reverse transcription using the Verso cDNA Kit (Thermo Fisher Scientific, Waltham, MA, USA). Relative amounts of gene transcripts were determined by real-time qPCR using TB green premix Ex Taq II (Takara Bio, Kusatsu, Shiga) and the Thermal Cycler Dice TP850 system (Takara). PCR was performed in duplicate for each sample, and the measured expression level of each gene was normalized to that of *Hprt1* or *Gapdh*. Supplementary Table S1 lists the sequence information for the primers used. All primer sets are for mouse genes.

### ChIP-qPCR

ChIP was performed as described previously^57^ with some modifications. Briefly, Dynabeads sheep anti-mouse immunoglobulin G (IgG) (Invitrogen) was used for immunoprecipitation in combination with an anti-SMAD1 antibody (Bio Matrix Research) or normal mouse IgG (Santa Cruz, Dallas, TX, USA) as a negative control. Supplemental Table S1 lists the primers used for qPCR.

### Luciferase reporter assay

Luciferase reporter constructs of mouse Atoh8 SBRs, SBR1 (GGCGTC) or SBR2 (GGCGCC), were described previously.^32^ Mutations in these motifs, GataTC for SBR1 and GaattC for SBR2, were also generated.^32^ The 6xOSE2 luciferase reporter plasmid was a kind gift from Dr. Toshihisa Komori (Nagasaki University, Japan). The Renilla luciferase reporter vector pGL4.74 (hRluc/TK) (Promega, Madison, WI, USA) was used as an internal control. Cells were seeded in triplicate in 24-well plates and transiently transfected with reporter luciferase plasmids with or without expression vectors. Dual luciferase assays were performed using a GloMax 96 microplate luminometer (Promega).

### Expression vectors

The human *ATOH8* gene (GenBank accession No. NM_032827.5) was amplified by PCR and subcloned into the pcDEF3-FLAG or −3 × FLAG plasmid. Deletion mutants were generated using PCR with specific primers with sites for restriction enzymes.^32^ The N-terminally Halo-tagged human ATOH8 pFN21A vector was purchased from the Kazusa DNA Research Institute (Kisarazu, Chiba, Japan). The V5-tagged LacZ pEF DEST51 vector (Invitrogen) or pcDNA3 plasmid was used as a mock control. The C-terminally V5-tagged mouse Runx2 plasmid has been previously described.^27^ Expression vectors were transfected using FuGENE6 (Promega) or Lipofectamine 3000 (Invitrogen).

Transfection experiments were approved by the Institutional Safety Committee for Recombinant DNA Experiments of Kagoshima University (S28032).

### In situ hybridization

Formalin-fixed paraffin-embedded (FFPE) samples of humeri from embryonic day E17.5 mice or tibiae from 8-week-old mice, decalcified using G-Chelate Mild solution (Genostaff, Tokyo, Japan), were subjected to ISH using an RNAscope 2.5 HD Reagent Kit (BROWN or RED; Advanced Cell Diagnostics, Newark, CA, USA) according to the manufacturer’s instructions. Specific probes against mouse *Atoh8*, as well as control probes against peptidylprolyl isomerase B (*Ppib*) (a housekeeping gene, positive control) and *DapB* (a bacterial gene, negative control), were obtained from Advanced Cell Diagnostics. Counterstaining was performed using hematoxylin solution.

### Immunohistochemistry (IHC)

The FFPE sections were deparaffinized and subjected to antigen retrieval by incubation with L.A.B. solution (Polysciences, Warrington, PA, USA). Endogenous peroxidase was inactivated by 3% hydrogen peroxide in methanol. The cell membrane was permeabilized with 0.2% Triton-X-100 in PBS. Nonspecific protein binding was blocked using CAS block (Thermo Fisher Scientific). The primary antibodies used in this study were mouse monoclonal anti-Runx2 (1:200, D130-3, MBL, Nagoya, Japan), rabbit polyclonal anti-Osterix (Osx) (1:500, ab22552, Abcam, Cambridge, UK), rabbit polyclonal anti-IBSP (Bsp) (1:50, LS-C190916, LSBio, Seattle, WA, USA), rabbit polyclonal anti-Osteocalcin (Ocn) (1:100, ab93876, Abcam), mouse monoclonal anti-RANKL (1:200, ab45039, Abcam), and rabbit polyclonal anti-Opg (1:100, ab183910, Abcam) antibodies. Normal rabbit IgG (sc-2027, Santa Cruz Biotechnology) and normal mouse IgG (sc-2025, Santa Cruz Biotechnology) served as negative controls for primary antibodies. Histofine Simple Stain MAX-PO (MULTI) (424151, Nichirei Biosciences, Tokyo, Japan), a mixture of anti-mouse and anti-rabbit peroxidase-conjugated secondary antibodies, was applied to detect primary antibody signals. Signals were visualized using DAB solution (415171, Nichirei Biosciences). Counterstaining was performed with Mayer’s hematoxylin solution. Images were captured using the BZ-X710/BZ-X700 microscope system (Keyence, Osaka, Japan).

### Atoh8-KO mice

Atoh8-null C57BL/6J mice have been described recently.^32^ A major part of exon 1 of *Atoh8*, including the initiating methionine codon, was deleted and replaced with a cassette encoding IRES-*lacZ* and neomycin resistance. *Atoh8* mutant mice were backcrossed onto C57BL/6 mice (Japan SLC, Shizuoka, Japan) for at least 12 generations.

All animal experiments were approved by the Institutional Animal Care and Use Committee of Kagoshima University (MD17021, MD17037, MD17070, and MD17085) and the University of Tokyo (Medicine-P16-140).

### Skeletal preparation

The skin and muscle of neonatal mice were removed and stained with alcian blue and alizarin red (Sigma-Aldrich) according to a standard protocol for skeletal preparation. Briefly, skeletal samples were fixed in 96% ethanol and stained with 0.015% alcian blue 8GX in a mixture solution of 96% ethanol/acetic acid (4:1), followed by dehydration in 100% ethanol. The dehydrated skeletons were immersed briefly in 1% potassium hydroxide (KOH), followed by staining with 0.001% alizarin red S in 1% KOH.

### μ-CT and histomorphometric analysis of bones

Femurs or lumbar vertebrae were fixed in 70% ethanol and subjected to μ-CT or histomorphometric analysis, respectively. μ-CT scanning was performed using the ScanXmate-A100S Scanner (Comscantechno, Yokohama, Kanagawa, Japan). 3D microstructural image data were reconstructed, and structural indices were calculated using TRI/3D-BON software (Ratoc System Engineering, Tokyo, Japan). Bone mineral was calculated using TRI/3D-BON-BMD-PNTM software (Ratoc System Engineering).

For histomorphometric analyses, lumbar vertebrae or tibiae were dehydrated and embedded in glycol methacrylate. Next, 3-μm-thick coronal sections were cut using a microtome and stained with toluidine blue or TRAP. Static parameters of bone formation and resorption were measured in a defined area between 0.3 mm and 1.2 mm from the growth plate using the OsteoMeasure bone histomorphometry system (Osteometrics, Decatur, GA, USA).

### Enzyme-linked immunosorbent assay (ELISA)

Quantitative determination of PINP and CTX-I was performed using a Rat/Mouse PINP EIA Kit (AC-33F, Immunodiagnostic systems, Tyne and Wear, UK) and a RatLaps CTX-I EIA Kit (AC-06F1, Immunodiagnostic systems), respectively, according to the manufacturer’s instructions.

### Alkaline phosphatase, tartrate‐resistant acid phosphatase, and von Kossa staining

Histochemical analysis of the ALP assay was performed using the ALP staining kit (85L-3R, Sigma-Aldrich) in accordance with the manufacturer’s instructions. Calcium deposition was visualized using the von Kossa method using 2.5% silver nitrate. Osteoclasts were visualized by TRAP staining. The cells were fixed and stained using the leukocyte acid phosphatase kit (387A, Sigma-Aldrich).

### RNA interference

Three independent stealth siRNAs (MSS291443, MSS291444, and MSS291445) against the mouse *Atoh8* gene were obtained from Invitrogen and combined as a mixture. Dharmacon siRNA ON-TARGETplus SMARTpool, a mixture of four independent siRNAs against mouse *Runx2* (L-064819-03), and the control siRNA were purchased from Thermo Fisher Scientific. The siRNAs were transfected into cells using Lipofectamine RNAiMax (Invitrogen).

### Immunoprecipitation and immunoblotting

COS-7 cells were transfected with expression plasmids overnight and incubated with or without the proteasome inhibitor MG132 (Merck, Kenilworth, NJ, USA) at 20 μmol·L^−1^ for 2 h. The cells were then lysed in NP-40 lysis buffer (150 mmol·L^−1^ NaCl, 1% NP-40, 50 mmol·L^−1^ Tris-HCl; pH 8.0) supplemented with aprotinin and phenylmethylsulfonyl fluoride (PMSF). The lysate was immunoprecipitated with an anti-FLAG M2 antibody (Sigma-Aldrich), followed by incubation with Dynabeads protein G (Invitrogen) according to the manufacturer’s instructions. Immunoprecipitated protein or input control protein was subjected to sodium dodecyl sulfate–polyacrylamide gel electrophoresis (SDS-PAGE) and polyvinylidene difluoride membrane transfer. Blots were incubated with an anti-FLAG M2 antibody or a horseradish peroxidase (HRP)-conjugated anti-V5 antibody (Invitrogen). An HRP-conjugated anti-mouse secondary antibody (Cell Signaling Technology, Danvers, MA, USA) was used to detect anti-FLAG antibody signals. The signals were developed by chemiluminescence.

### Statistical analysis

The results are expressed as the mean ± standard deviation of at least three independent experiments. Statistical comparisons between various treatments were performed using unpaired Student’s *t* test. *P* < 0.05 was considered statistically significant.

## Supplementary information

Supplementary Table S1

Supplementary Figure S1

Supplementary Figure S2

Supplementary Figure S3

Supplementary Figure S4
