# Supplementary figures and images for: BMP-induced Atoh8 attenuates osteoclastogenesis by suppressing Runx2 transcriptional activity and reducing the Rankl/Opg expression ratio in osteoblasts
Source: Bone Res. 2020 Sep 2;8:32. doi: 10.1038/s41413-020-00106-0 (PMC7463266; doi:10.1038/s41413-020-00106-0)

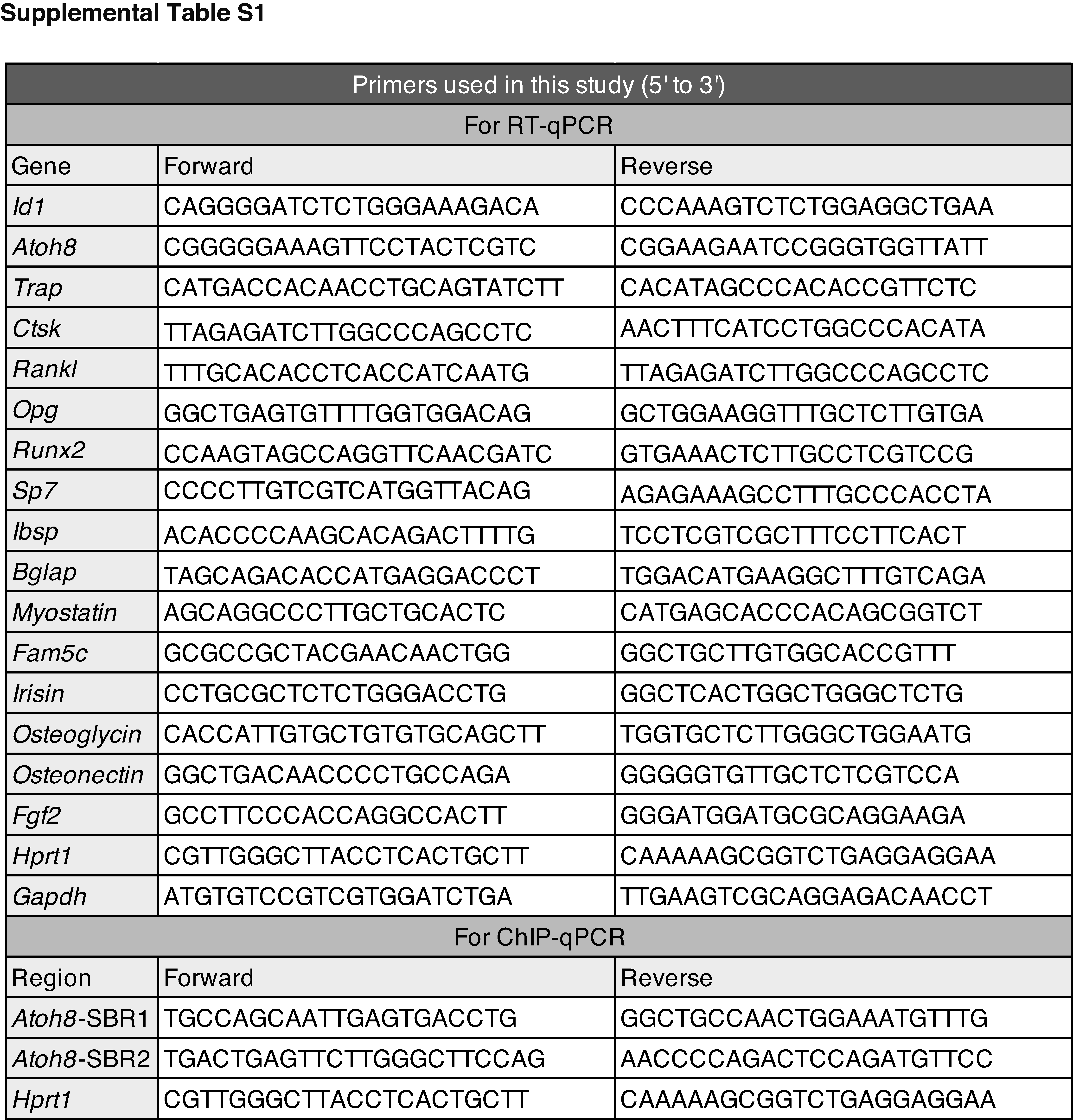

Supplement: Supplementary file 1 — Supplementary Table S1 [file 41413_2020_106_MOESM1_ESM.jpg]

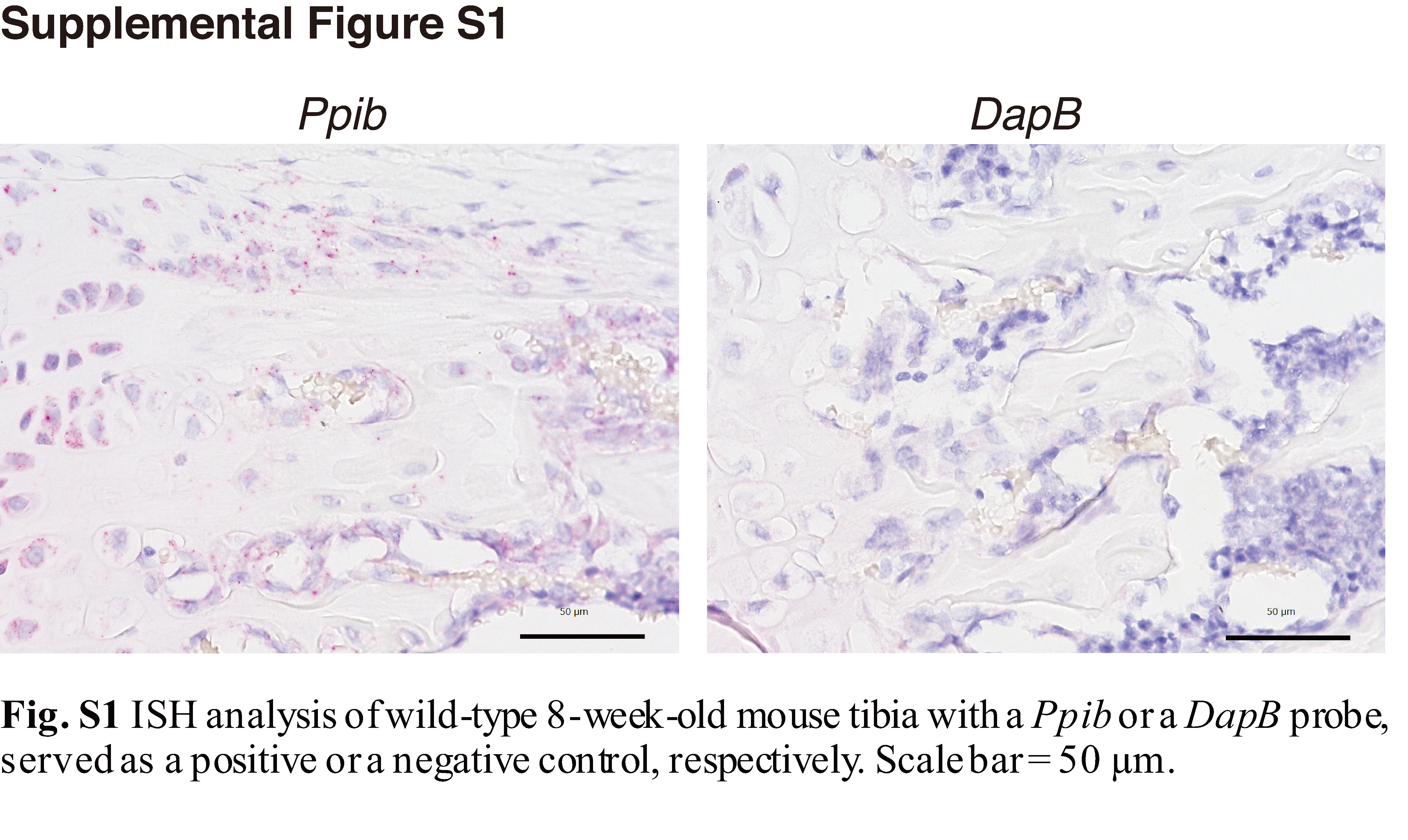

Supplement: Supplementary file 2 — Supplementary Figure S1 [file 41413_2020_106_MOESM2_ESM.jpg]

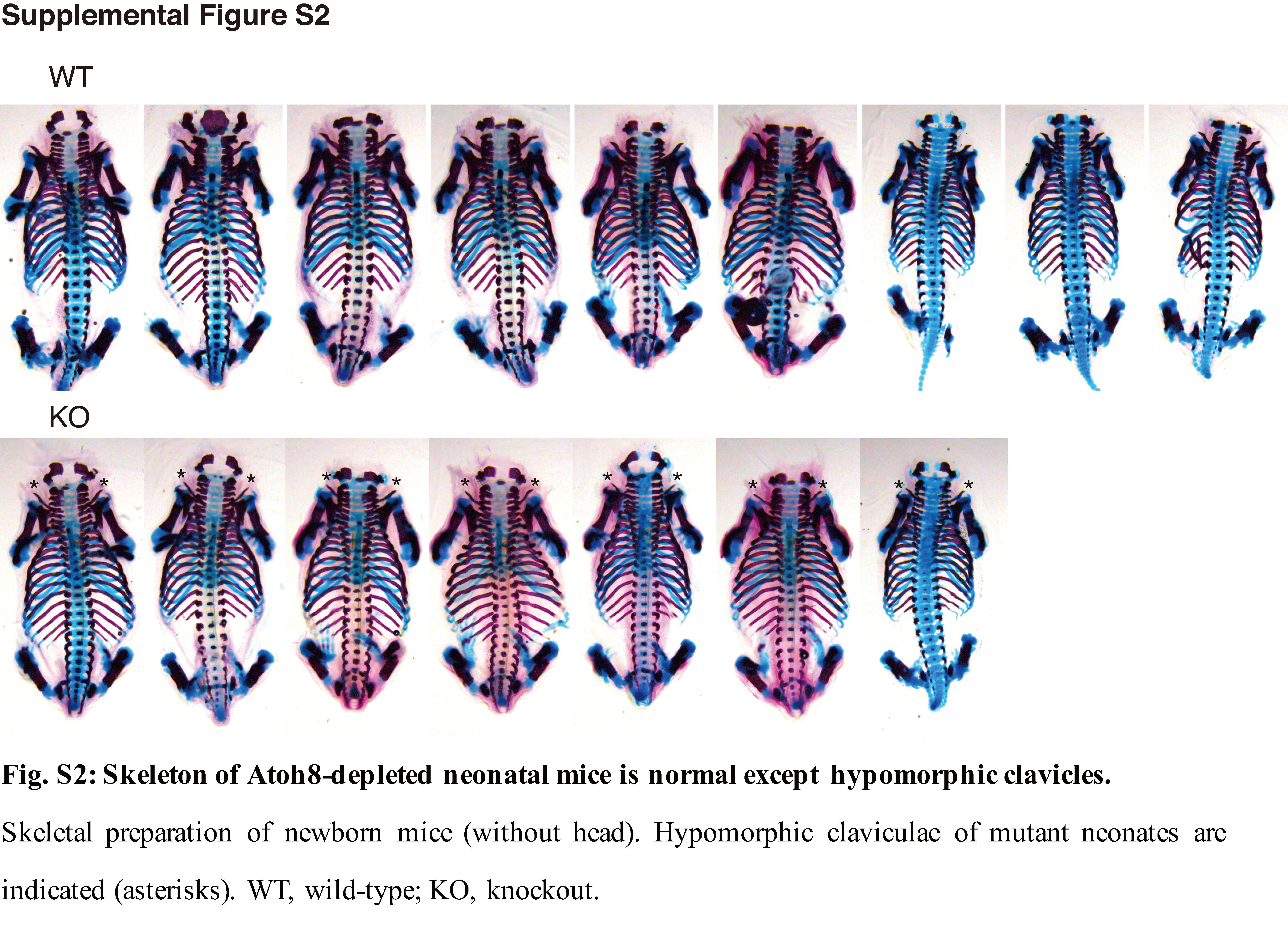

Supplement: Supplementary file 3 — Supplementary Figure S2 [file 41413_2020_106_MOESM3_ESM.jpg]

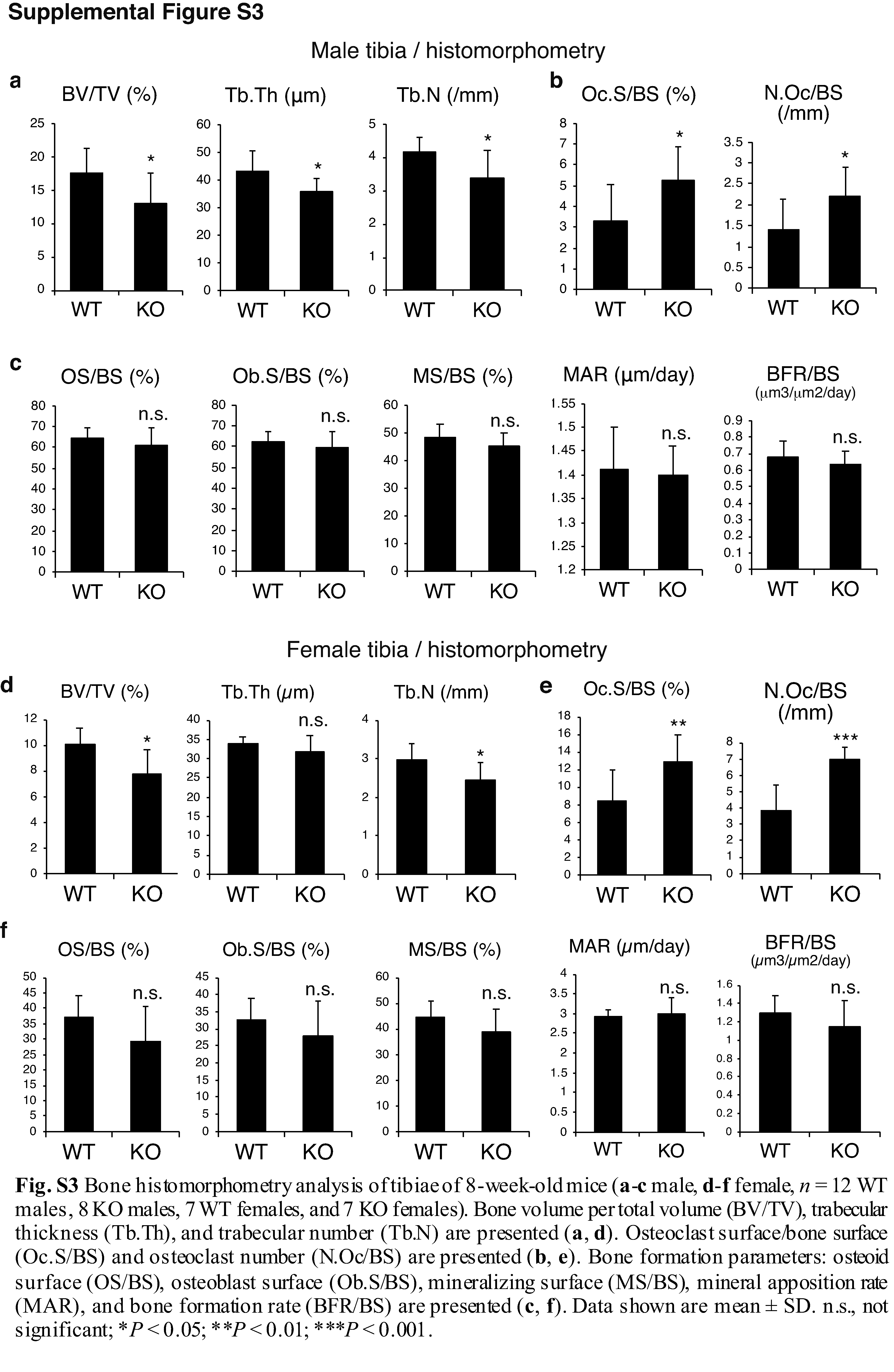

Supplement: Supplementary file 4 — Supplementary Figure S3 [file 41413_2020_106_MOESM4_ESM.jpg]

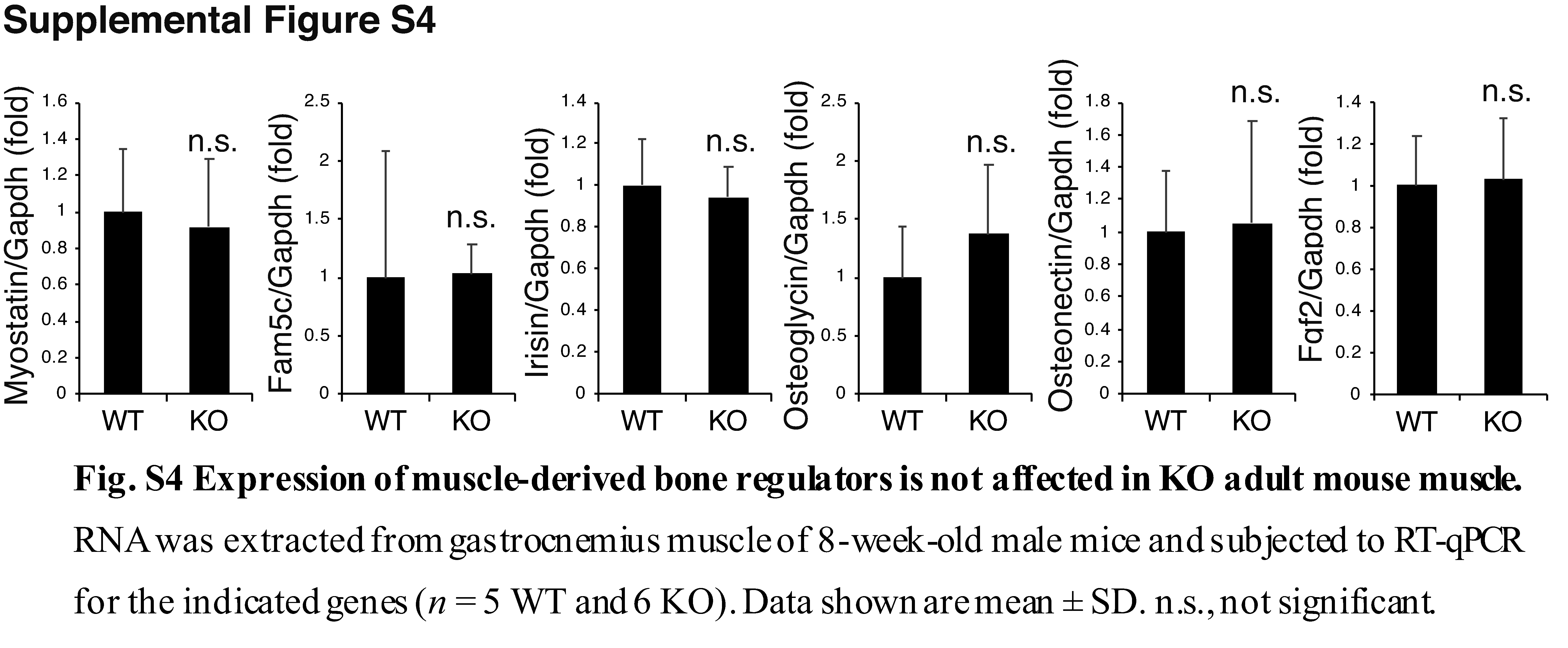

Supplement: Supplementary file 5 — Supplementary Figure S4 [file 41413_2020_106_MOESM5_ESM.jpg]
